# *ETV6*::*ABL1* fusion: from overlooked minor clone in myeloproliferative neoplasm to major player in leukemic transformation

**DOI:** 10.1007/s00428-024-03881-x

**Published:** 2024-07-27

**Authors:** Hyun-Woo Lee, Min-Seung Park, Boram Kim, Chul Won Jung, Hee-Jin Kim, Hyun-Young Kim

**Affiliations:** 1 Department of Laboratory Medicine and Genetics, Samsung Medical Center, Sungkyunkwan University School of Medicine, Seoul, Republic of Korea; 2 Division of Hematology-Oncology, Department of Medicine, Samsung Medical Center, Sungkyunkwan University School of Medicine, Seoul, Republic of Korea

**Keywords:** *ETV6*::*ABL1*, Myeloid/lymphoid neoplasms with eosinophilia and tyrosine kinase gene fusions, Myeloproliferative neoplasm, Eosinophilia

## Abstract

The *ETV6*::*ABL1 *fusion defines a subgroup of myeloid/lymphoid neoplasms with eosinophilia and tyrosine kinase gene fusions. We report a case of extramedullary involvement and leukemic transformation in myeloproliferative neoplasm (MPN), where *ETV6*::*ABL1 *was initially overlooked but later detected in the blast phase. *ETV6*::*ABL1 *burden was very low during the MPN phase but increased substantially during the blast phase. This correlation between *ETV6*::*ABL1 *burden and disease phenotype indicated that an immature leukemic clone is the sole carrier of *ETV6*::*ABL1*, suggesting that *ETV6*::*ABL1 *is not the primary driver of the MPN phase. Moreover, only the blast phase revealed somatic mutations in *RUNX1 *and *STAG2*, or complex karyotype, while the MPN phase revealed no molecular and cytogenetic abnormalities. Therefore, it remains uncertain whether the small clone of *ETV6*::*ABL1 *influenced the manifestation of MPN or if another underlying driver was responsible for the MPN phase, necessitating further research.

## Introduction

The *ETV6*::*ABL1* fusion was recently included in the genetic abnormalities defining the disease category of myeloid/lymphoid neoplasms with eosinophilia and tyrosine kinase gene fusions (MLN-TK) [1, 2]. To date, *ETV6*::*ABL1* has been reported on various hematological malignancies, including B/T-acute lymphoblastic leukemia, acute myeloid leukemia (AML), and myeloproliferative neoplasm (MPN) [3, 4]. The MLN-TK with *ETV6*::*ABL1* shares common clinicopathological features with chronic myeloid leukemia, such as eosinophilia and response to tyrosine kinase inhibitor (TKI) therapy, due to the structural and functional similarity of the fusion proteins [5–8].

Herein, we present a case of MLN-TK with *ETV6*::*ABL1*, which was identified after the extramedullary involvement and leukemic progression of MPN, to share a unique disease manifestation.

## Materials and methods

### Study case

A case of MLN-TK with *ETV6*::*ABL1* was obtained at our institution. The clinical and laboratory information of the patient was collected from electronic medical records. This study was approved by the Institutional Review Board of Samsung Medical Center (2023–02-092), and the requirement for informed consent was waived.

### Cytogenetic study

A conventional cytogenetic study was performed on heparinized bone marrow (BM) samples using a standard G-banding technique following short-term culturing without mitogen, and at least twenty metaphases were analyzed for karyotyping. A fluorescence in situ hybridization (FISH) study was performed on the BM aspiration slide using the Vysis LSI *BCR*/*ABL1* Dual Color, Dual Fusion Translocation probe (Abbott Laboratories, Abbott Park, IL, USA), *ETV6*/*RUNX1* ES Dual Color Translocation probe (Abbott), 4q12 Tri-Color Rearrangement probe for *FIP1L1*-*PDGFRA* region (Abbott), *PDGFRB* Dual-Color Break Apart probe (Abbott), and XL *FGFR1* Break Apart probe (MetaSystems, Altlussheim, Germany) according to the manufacturer’s instructions, and at least 200 interphase cells were analyzed.

### Molecular genetic study

Genomic DNA and RNA were extracted from BM aspirate samples using a Promega DNA extraction kit (Promega, Madison, WI, USA) and QIAamp RNA Blood Mini kit (Qiagen, Hilden, Germany), respectively.

Multiplex reverse transcription PCR (RT-PCR) was performed with HemaVision (DNA Technology, Aarhus, Denmark) according to the manufacturer’s instructions. In-house RT-PCR for *ETV6*::*ABL1* was performed using the 5′ *ETV6* and 3′ *ABL1* primers (Forward primer for *ETV6* exon 5, 5′-TCAAACAGTCCAGGCTCTCC-3′; Reverse primer for *ABL1* exon 2, 5′-CCACTGGCCACAAAATCATA-3′). PCR products were subjected to direct sequencing by using the BigDye Terminator Cycle Sequencing Ready Reaction Kit (Applied Biosystems, Foster City, CA, USA) on ABI Prism 3130xl Genetic Analyzer (Applied Biosystems).

For targeted next-generation sequencing (NGS) for 49 myeloid neoplasm-related genes, sequencing libraries were prepared using the IDT xGen pre-designed/custom probes (Integrated DNA Technologies, Coralville, USA), and sequencing was performed on the NextSeq 550Dx (Illumina, San Diego, CA, USA). Reads were aligned using the BWA-MEM to the GRCh37/hg19 human reference genome, and variant calling, annotation, and analysis were carried out using an in-house pipeline.

## Results

### Case presentation

**Fig. 1:**
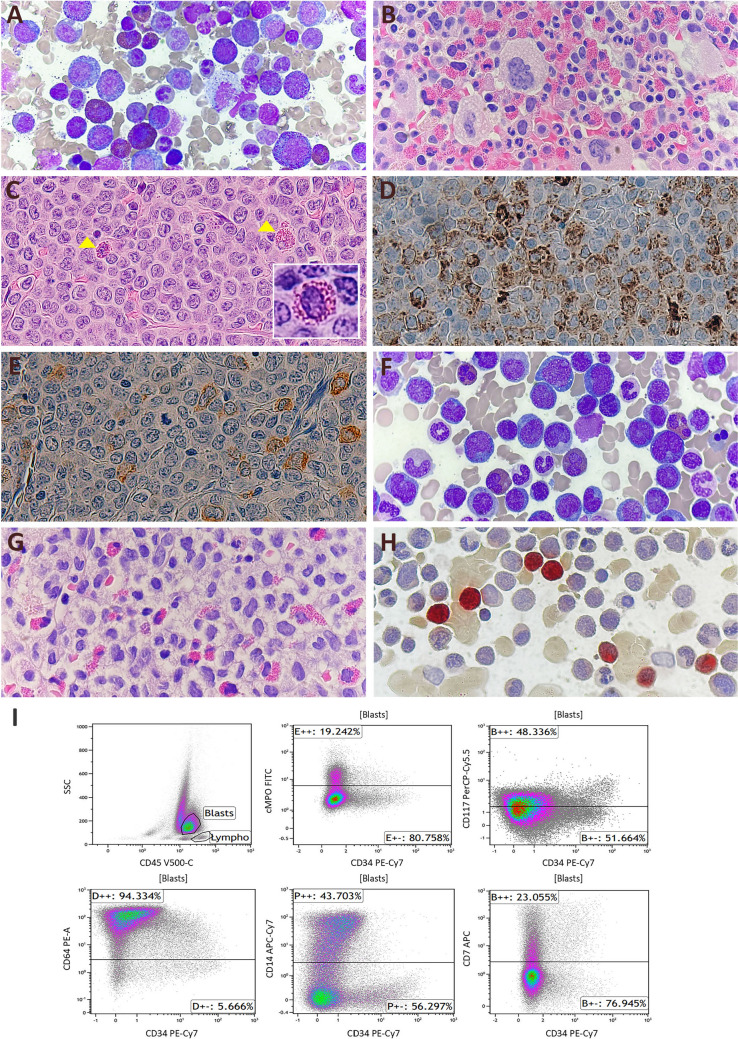
Bone marrow (BM) aspiration smear and biopsy, cervical lymph node (LN) biopsy, and flow cytometric analysis. **A**, **B** At initial diagnosis, BM aspiration smear (**A** Wright-Giemsa [W&G] stain, × 400) and biopsy (**B** hematoxylin & eosin [H&E] stain, × 400) revealed 90% hypercellular marrow with a prominent increase of granulocytes, eosinophils, and megakaryocytes. **C**–**I** At the time of leukemic transformation, cervical LN biopsy (**C** H&E stain, × 400) revealed myeloid sarcoma with monocytic differentiation, accompanied by a small number of eosinophils (arrowhead): Approximately half of the cells were positive for lysozyme (**D**, × 400), while only a few cells exhibited weak positivity for myeloperoxidase [MPO] (**E**, × 400) immunohistochemistry (IHC). BM aspiration smear (**F** W&G stain, × 400) and biopsy (**G** H&E stain, × 400) also showed increased blasts, which were positive on alpha-naphthyl butyrate esterase cytochemical stain, exhibiting a reddish-brown reaction throughout the entire cytoplasm and, in cases of strong reactivity, over the nuclei (**H**, × 400). Flow cytometric analysis (**I**) demonstrated CD34-negative blasts with expression of cMPO^partial^, CD117^dim^, CD64^bright^, CD14^partial^, and CD7^partial^

A 29-year-old man visited our hospital with leukocytosis persisting over 12 months. Complete blood count (CBC) showed leukocytosis with left-shifted maturation and eosinophilia: white blood cells (WBC), 30.63 × 10^9^/L (with 4% myelocytes/metamyelocytes and 9% eosinophils); hemoglobin (Hb), 14.8 g/dL; and platelets, 378 × 10^9^/L. BM aspiration smear and biopsy section showed hypercellularity with increased trilineage hematopoiesis and eosinophilia without dysplasia or fibrosis, similar to chronic myeloid leukemia (Fig. 1A, B). Chromosomal analysis revealed a normal karyotype. There was no evidence of eosinophilia-related cytogenetic alterations on the FISH study for *PDGFRA*, *FIP1L1*, *PDGFRB*, and *BCR*/*ABL1*. Targeted NGS including *JAK2*, *CALR*, and *MPL* also identified no mutation, which led to the initial diagnosis of MPN, Unclassifiable. Empirically, imatinib was attempted for 1 month, but no significant treatment response was observed. The 2nd BM study performed 10 months later also showed hypercellular marrow and eosinophilia, similar to the 1st BM study.

**Fig. 2:**
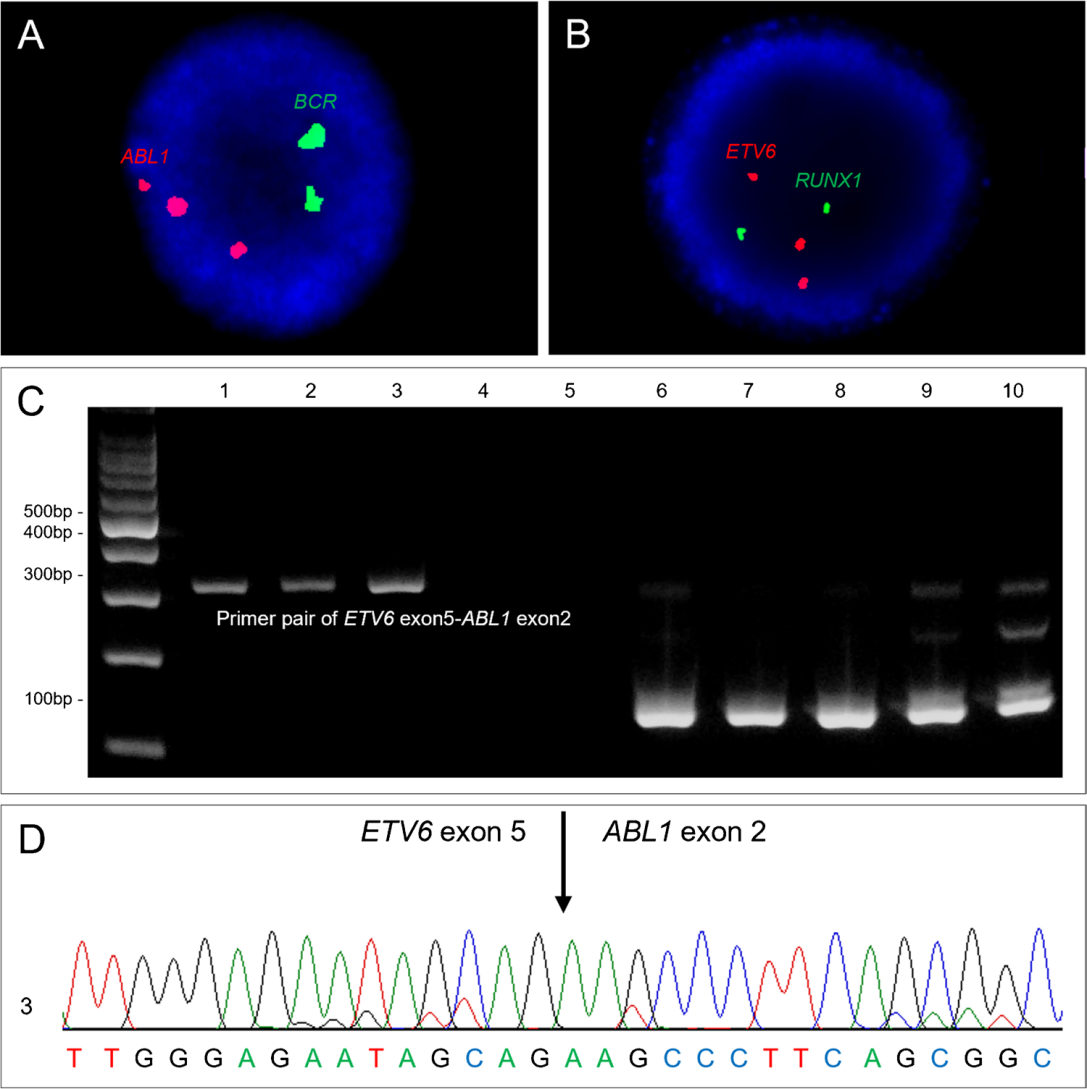
Fluorescence in situ hybridization (FISH) and molecular genetic study for *ETV6*-*ABL1* rearrangement. **A**, **B** FISH analysis using *BCR*/*ABL1* (**A**) and *ETV6*/*RUNX1* probes (**B**) revealed three *ABL1* signals and three *ETV6* signals, respectively, at the time of leukemic transformation. **C** Reverse transcription PCR for *ETV6*::*ABL1* fusion transcript was positive in lanes 1–3 and negative in lanes 4–5 (lane 1, at the time of initial diagnosis of myeloproliferative neoplasm [MPN]; lane 2, follow-up of MPN; lane 3, at the time of leukemic transformation; lane 4, follow-up after induction chemotherapy; and lane 5, follow-up after allogenic hematopoietic stem cell transplantation [target amplicon size, 298 bp]). Lanes 6–10 indicate internal control using *GAPDH* [target amplicon size, 131 bp]) for each sample. **D** Sanger sequencing revealed the breakpoint of fusion transcript of *ETV6* exon 5 and *ABL1* exon 2 on sample of lane 3

The patient developed painful neck swelling 5 months after the 2nd BM study and an excisional biopsy on the cervical lymph node (LN) confirmed myeloid sarcoma (Fig. 1C–E). CBC showed WBC of 141 × 10^9^/L with 4% blasts, left-shifted maturation and eosinophilia, Hb of 8.0 g/dL, and platelet of 21 × 10^9^/L. BM aspiration smear showed 48% blasts and positive cytochemical staining on alpha-naphthyl butyrate esterase (Fig. 1F–H). Flow cytometry analysis revealed that the blasts were positive for cytoplasmic MPO^partial^, CD14^partial^, CD64, CD13, CD33, CD117^dim^, CD7^partial^, and HLA-DR; and negative for cytoplasmic and surface CD3, CD10, CD19, cytoplasmic CD22 and CD79a, consistent with AML with monocytic differentiation (Fig. 1I). Chromosomal analysis showed normal karyotype, but multiplex RT-PCR revealed *ETV6*::*ABL1*. A FISH study using probes for *ETV6*/*RUNX1* and *BCR*/*ABL1* showed 78.0% and 76.0% of cells with three *ETV6* and *ABL1* signals, respectively, indicative of *ETV6*/*ABL1* rearrangement (Fig. 2A, B). NGS identified three mutations on *RUNX1* and *STAG2*. After the leukemic progression, the patient received induction chemotherapy of idarubicin and cytarabine. Consolidation chemotherapy and allogenic hematopoietic stem cell transplantation (HSCT) followed, and morphologic remission was attained a month after HSCT.

A follow-up CBC, performed 5 months after HSCT, showed 36% blasts, and a concurrently done BM study revealed relapse. Chromosomal analysis showed complex karyotype: 46,XY,t(5;18)(q23;q22),t(9;17)(q32;q25),t(10;14)(q24;q11.2)[17]/46,Y,t(X;12)(q13;p13)[2]//46,XX[1]. FISH analysis with probes for *ETV6*/*RUNX1* and *BCR*/*ABL1* detected 80.0%, 80.0%, and 77.0% of cells with three *ETV6, RUNX1*, and *ABL1* signals, respectively. Multiplex RT-PCR revealed the reappearance of *ETV6*::*ABL1*, but no mutation was detected on NGS. After induction chemotherapy, the patient developed neutropenic fever and pneumonia and expired within a month.

### ETV6::ABL1 fusion

RT-PCR for *ETV6*::*ABL1* was retrospectively performed, confirming the existence of *ETV6*::*ABL1* not only at the time of leukemic transformation but also at the initial diagnosis and during follow-up period of MPN (Fig. 2C). Direct sequencing confirmed the fusion of *ETV6* exon 5 and *ABL1* exon 2 (Fig. 2D). On the other hand, *ETV6*::*ABL1* was not detected by RT-PCR after induction chemotherapy and allogenic HSCT. The patient’s BM findings and test results are summarized in Table 1 in a chronological order.

**Table 1:** Summary of bone marrow and cytogenetic and molecular genetic test results

BM number	#1	#2	#3	#4	#5	#6	#7
Time point	Initial Dx	10 mo. after Dx (with 1 mo. of imatinib Tx)	15 mo. after Dx	1 mo. after induction CTx	1 mo. after allogenic HSCT	3 mo. after allogenic HSCT	5 mo. after allogenic HSCT
Disease phenotype based on BM aspirate and biopsy	MPN	MPN	BP^a^ (1st AML)	MR	MR	MR	BP (2nd AML)
Eosinophil count (× 10^9^/L) in peripheral blood	2.76	7.03	2.82	0	0.02	0	0
Blasts (%)^b^	< 5%	< 5%	48%	< 5%	< 5%	< 5%	67%
Somatic mutation (VAF, %)^b^	No variant detected	No variant detected	*RUNX1*:c.422_423insCC (4.3%);c.508 + 1_508 + 2insGCTTGGAGTGGAAGAGG (18.0%), *STAG2*:c.143del (43.7%)	NT	NT	NT	No variant detected
Karyotype^b^	46,XY[20]	46,XY[20]	46,XY[20]	46,XY[20]	//46,XX[20]^c^	//46,XX[20]^c^	46,XY,t(5;18)(q23;q22),t(9;17)(q32;q25),t(10;14)(q24;q11.2)[17]/46,Y,t(X;12)(q13;p13)[2]//46,XX[1]^c^
Proportion (%) of cells with three *ABL1* signals on *BCR*/*ABL1* FISH^b^	0%	NT	76.0%	0.5%	2.0%	1.0%	77.0%
*ETV6*::*ABL1* fusion transcript on RT-PCR^b^	Detected	Detected	Detected	Not detected	Not detected	NT	Detected

*Dx* diagnosis, *mo.* month, *Tx* treatment, *CTx* chemotherapy, *HSCT* hematopoietic stem cell transplantation, *BM* bone marrow, *MPN* myeloproliferative neoplasm, *AML* acute myeloid leukemia, *BP* blast phase, *MR* morphologic remission, *VAF* variant allele frequency, *NT* not tested, *FISH* fluorescence in situ hybridization, *RT-PCR* reverse transcription PCR

^a^Accompanied by extramedullary leukemic involvement

^b^Test results based on BM aspirate specimen

^c^This patient received a sex-mismatched allogeneic HSCT

## Discussion

The *ETV6*::*ABL1* fusion is often cryptic due to similar G-banding patterns of chromosomal bands 9q34 and 12p13, making it difficult to detect with conventional chromosomal analysis [9]. Also, *ETV6*::*ABL1* could result from a cryptic insertion creating an in-frame fusion gene and this small insertion could be missed when FISH with either *BCR*/*ABL1* or *ETV6*/*RUNX1* probes is used [4, 10]. Therefore, the combination of both probes has been suggested to reliably detect the *ETV6*::*ABL1* fusion. In our case, *ETV6*::*ABL1* was detected during the blast phase using multiplex RT-PCR and subsequently confirmed and semi-quantified by FISH.

Given that *ETV6*::*ABL1* has been predominantly reported in both MPN and AML, we retrospectively assessed and confirmed the presence of *ETV6*::*ABL1* during the initial MPN phase using RT-PCR. However, considering that the initial *BCR*/*ABL1* FISH study performed on BM aspiration smear was normal, it suggested that *ETV6*::*ABL1* was present at a very low burden during the MPN phase. A previous study showed that *ETV6*::*ABL1* was detected on hematopoietic stem cells, monocytes, and granulocytes, but not on mature lymphocytes [6]. On the contrary, to explain our findings, we had to speculate that immature leukemic clone serves as the sole carrier of *ETV6*::*ABL1*. The immature leukemic cells harboring *ETV6*::*ABL1* stayed as a minor clone during the MPN, but during the blast phase, they proliferated uncontrollably, increasing the burden of *ETV6*::*ABL1*. Interestingly, in our case, *ETV6*::*ABL1* did not appear to be the primary driver of the MPN phase. However, no mutations were observed in the two separate targeted NGS studies conducted using BM aspirates during the MPN phase. Therefore, it remains unclear whether the small clone of *ETV6*::*ABL1* influenced the manifestation of MPN or if there was another underlying driver affecting the preferential expansion of granulocytes, eosinophils, and megakaryocytes.

Eosinophilia, a morphological hallmark of MLN-TK with *ETV6*::*ABL1* [6, 11, 12], persisted throughout the disease progression in our case, from the MPN phase to the 1st blast phase (Table 1). Previous studies have also shown the presence of eosinophilia in all MPN and AML cases with *ETV6*::*ABL1* [4, 6, 8, 13]. However, eosinophilia was not prominent in the 2nd blast phase, where clonal evolution may have contributed to this non-eosinophilic phenotype.

On the diagnostic side, we encountered a challenge where the *ETV6*::*ABL1* was obscured during the MPN phase, only to be detected late during the blast phase, which significantly delayed early diagnostic suspicion of MLN-TK and its corresponding treatment. This appeared to be primarily due to the low sensitivity of FISH analysis rather than a cryptic structural variation, a well-known cause of the false negative result in FISH [8, 10]. Therefore, an RNA-based study capable of sensitively detecting *ETV6*::*ABL1* should be used for diagnostic evaluation, especially in patients with MPN featuring eosinophilia and lacking the *BCR*::*ABL1*, considering clonal burden and disease phenotype in the interpretation of results.

Prognosis of MLN-TK with *ETV6*::*ABL1* is strongly associated with disease stage and response to TKI. The overall survival is longer in the chronic phase than the blast phase, and 2nd generation TKIs have been reported to be more effective than imatinib in inducing durable remission [14]. Zaliova et al. reported the poor outcome of ALL and AML with *ETV6*::*ABL1* [4]. As genomic complexity increases during the blast crisis, patients who were diagnosed at this advanced stage hardly survived despite TKI therapy, very similar to our case. The 1st blast phase in our case revealed the acquisition of *RUNX1* and *STAG2* mutations, which is associated with a poor prognosis [15]. In the 2nd blast phase, complex cytogenetic abnormalities emerged and the acquired mutations disappeared, suggesting increased genomic instability and clonal evolution, which may have led to dismal prognosis.

In conclusion, we presented a unique case of MLN-TK with *ETV6*::*ABL1* in which *ETV6*::*ABL1* was not the primary driver during the MPN phase, remaining as a minor clone, but ultimately progressed to AML. The underlying mechanism of this phenomenon has not been elucidated and further research is needed.

## Data Availability

All data generated or analyzed during this study are included in this published article.
