# Correction to: *ETV6*::*ABL1* fusion: from overlooked minor clone in myeloproliferative neoplasm to major player in leukemic transformation

**DOI:** 10.1007/s00428-024-03893-7

**Published:** 2024-08-13

**Authors:** Hyun‑Woo Lee, Min‑Seung Park, Boram Kim, Chul Won Jung, Hee‑Jin Kim, Hyun‑Young Kim

**Affiliations:** 1 Department of Laboratory Medicine and Genetics, Samsung Medical Center, Sungkyunkwan University School of Medicine, Seoul, Republic of Korea; 2 Division of Hematology‑Oncology, Department of Medicine, Samsung Medical Center, Sungkyunkwan University School of Medicine, Seoul, Republic of Korea

**Correction to: Virchows Archiv**

10.1007/s00428-024-03881-x

In the original published version of the article, the abstract and keywords were omitted due to an error by the publisher.

The original article has been corrected.

